# Textile Dye Decolorizing Synechococcus PCC7942 Engineered With CotA Laccase

**DOI:** 10.3389/fbioe.2018.00095

**Published:** 2018-07-12

**Authors:** Yuanmei Liang, Juan Hou, Ying Liu, Yifan Luo, Jie Tang, Jay J. Cheng, Maurycy Daroch

**Affiliations:** ^1^School of Environment and Energy, Shenzhen Graduate School, Peking University, Shenzhen, China; ^2^College of Life Sciences and Oceanography, Shenzhen University, Shenzhen, China; ^3^School of Pharmacy and Bioengineering, Chengdu University, Chengdu, China; ^4^Department of Biological and Agricultural Engineering, North Carolina State University, Raleigh, NC, United States

**Keywords:** DNA assembly, laccase, dye decolorization, cyanobacterium, microbial cell factory

## Abstract

Cyanobacteria are prokaryotic phototrophs capable of achieving high cellular densities with minimal inputs. These prokaryotic organisms can grow using sunlight as energy source and carbon dioxide as carbon source what makes them promising candidates as microbial cell factories for the production of numerous compounds such as chemicals, fuels, or biocatalysts. In this study, we have successfully designed and constructed using synthetic biology approach two recombinant strains of *Synechococcus elongatus* PCC7942 for heterologous expression of the industrially relevant *Bacillus subtilis* CotA laccase. One of the strains (PCC7942-NSI-CotA) was constructed through integration of the laccase gene into neutral site I of the cyanobacterial genome whilst the other (PCC7942-NSII-CotA) targeted neutral site II of the genome. Of the two strains the one with CotA laccase integrated in neutral site II (PCC7942-NSII-CotA) was superior in terms of growth rate and enzymatic activity toward typical laccase substrates: ABTS [2,2-azino-bis (3-ethylbenzothiazoline-6-sulfonate)] and syringaldazine. That may suggest that two of the traditionally used neutral sites of *S. elongatus* PCC7942 are not equally suitable for the expression of certain transgenes. The PCC7942-NSII-CotA produced protein was capable of decolourising three classes of dyes namely: anthraquinonic-, azo-, and indigoid-type over 7 days of incubation making the strain a potentially useful microbial cell factory for the production of broad-spectrum biodegradation agent. Interestingly, presence of additional synthetic redox mediator ABTS had no effect on the degradation of these dyes.

## Introduction

Cyanobacteria are considered as promising candidates for microbial cell factories for the production of numerous compounds such as chemicals, fuels, or biocatalysts due to their good growth using carbon dioxide and sunlight and amenability to genetic engineering (Angermayr et al., 2009; Pisciotta et al., 2010). Protein production using cyanobacteria can have many advantages over traditional fermentation methods. Firstly, cyanobacteria can utilize CO_2_ from waste or industrial sources instead of glucose or other reduced carbon source; secondly, they use simple inorganic medium; thirdly, the energy for metabolic processes is provided by light harvesting, and last but not least they have relatively tractable tools for genetic engineering when compared to eukaryotic algae (Heidorn et al., 2011). Moreover, traditional methods of protein production often require energy intensive medium sterilization to prevent microbial contamination. Cyanobacteria are less prone to contamination due to lack of readily available carbon source. Genetic engineering of cyanobacteria has recently demonstrated photosynthetic production of many chemicals including ethanol (Deng and Coleman, 1999), lactic acid (Angermayr et al., 2012), isopropanol (Kusakabe et al., 2013; Hirokawa et al., 2015), 1-butanol (Atsumi et al., 2009), fatty acids (Liu et al., 2011; Ruffing and Jones, 2012; Ruffing, 2014), and so on.

Laccases (EC 1.10.3.2) are multi-copper oxidative enzymes, able to catalyse redox reactions involving transfer of four electrons from the substrate (phenols, polyphenols, and anilines) to the terminal electron acceptor (O_2_) to yield water as a by-product (Thurston, 1994; Paice et al., 1995; Huang et al., 1999; Rivera-Hoyos et al., 2013). Since laccases utilize exclusively molecular oxygen as a co-factor for catalysis it makes them potentially superior to other oxidoreductases that rely on less abundant compounds such as H_2_O_2_. Owing to their broad substrate specificities and environment-friendliness, laccases are promising oxidoreductases with extensive applications. In recent years they found use in following areas: bioremediation, synthetic chemistry, delignification of plant biomass for paper industry, cellulosic ethanol biofuels, and textile finishing (Lloret et al., 2012; Abdel-Hamid et al., 2013; Fernández-Fernández et al., 2013; Daroch et al., 2014; Mogharabi and Faramarzi, 2014; Viswanath et al., 2014). Decolorization of textile dye effluents has been the widest application of laccases because of their ability to catalyse breakdown of chromophore group compounds of azo- and anthraquinonic-reactive dyes. Their application for waste streams containing high concentrations of textile dyes, such as textile wastewater is promising alternative to conventional processes such as adsorption, chemical oxidation or so-called advanced oxidation processes (Holkar et al., 2016), could offer a lot benefits like eco-friendliness, cost-competitiveness, yield non-hazardous metabolites, lower sludge production, and water consumption to the environment (Hayat et al., 2015).

To fully explore potential of laccases for such a large scale low cost process a cheap and effective protein expression system is required. Unlike most oxidoreductases that are known to be notoriously difficult to express in heterologous hosts; laccases have been already successfully expressed in filamentous fungi, plants, yeast, and even bacteria (Ward, 2012; Bleve et al., 2014; Brander et al., 2014). However, to date no attempts to produce laccases in photosynthetic microorganisms like algae or cyanobacteria have been reported, which is potentially very promising approach taking into consideration co-factor requirement of the enzyme (molecular oxygen). Expression of a laccase enzyme in a photosynthetic host is likely to create an effect of synergy between products of photosynthesis and the co-factor requirement of the protein enhancing its potential as a sustainable biocatalyst when used in conjunction with photosynthesising cells.

In this article the utilization of cyanobacterium *Synechococcus elongatus* PCC7942 as a microbial cell factory to produce *B. subtilis* CotA laccase is described. Full length CotA gene was synthesized with codon preference of the host strain and assembled into two integrative vectors pCV0062 and pCV0063 targeting neutral sites NSI (Bustos and Golden, 1992) and NSII (Andersson et al., 2000) of *S. elongatus* chromosome, respectively. Recombinant integrative plasmids were transformed into *S. elongatus* PCC7942, and their successful integration was confirmed by PCR reactions. Fully segregated lines have been obtained through repetitive subcultures and the two strains were tested against the PCC7942 wild type using two typical indicator colorimetric substrates of laccases, ABTS and syringaldazine, proving functionality of both PCC7942-CotA transformants. Meanwhile, the ability of recombinant strain PCC7942-NSII-CotA to decolourise ten common textile dyes was also tested and proved positive.

## Materials and methods

### Strains and culture conditions

*Escherichia coli* strains DB3.1 and DH5α were purchased from Tiangen (China) and grown in LB medium at 37°C for sub-cloning procedures. Transformants of *E. coli* were propagated in LB medium with streptomycin (50 μg ml^−1^). *S. elongatus* PCC7942 was purchased from Institut Pasteur Cyanobacterial Culture Collection (France) and grown photoautotrophically is shaking incubator (100 rpm) using 200 ml conical flasks containing 100 ml of BG11 medium (Waterbury and Stanier, 1981) at 28°C under constant illumination with fluorescent white light of an intensity of 70 μmol m^−2^ s^−1^. *S. elongatus* PCC7942 transformants were initially selected on BG11 medium solidified with agar (15 g l^−1^) containing streptomycin a final concentration of 5 μg ml^−1^ (Sigma). For segregation and shake flask cultivation of transgenic strain the growth medium was supplemented with streptomycin to a final concentration of 50 μg ml^−1^. The growth of *S. elongatus* PCC7942 was monitored with spectrophotometer by measuring increase of optical density (OD) at 730 nm.

### Synthesis of laccase gene and construction integrative plasmids

Amino acid sequence of CotA laccase deposited at National Center for Biotechnology Information with the accession number BAA22774 was synthesized using codon bias of *S. elongatus* PCC7942 strain by GENEWIZ (China) and inserted into pUC57-Amp vector to generate pUC57-Amp-CotA vector. PCR primers were designed to contain 15 bp 5′and 3′ terminal overhangs homologous to the arms of the integrative vector. The full-length laccase gene containing overhangs was amplified from pUC57-Amp-CotA with Phusion *Pfu* DNA polymerase (NEB, USA) and CotA-veF, CotA-veR (Table 1) according to manufacturer's instructions. Resultant PCR product was purified, and sub cloned into the pJET1.2 Cloning Vector (Thermo Fisher Scientific, USA) and confirmed by sequencing by BGI Shenzhen (China).

**Table 1:** PCR primers used in this study.

**Primer name**	**Sequence 5^′^ → 3^′^**	**Annealing temperature**	**Amplicon length**	**Reference**
CotAF	ATGCCTGCAGGTCGACGATA	52	1539 bp	This study
CotAR	TTATTTATGAGGATCAGTAATATCCATAGGT			
CotA-veF	ATAACCCAGGGATTTATGCCTGCAGGTCGACGAT	58	1569 bp	This study
CotA-veR	GGAGCTCCTTCATTTTTATTTATGAGGATCAGTAATATCCA			
NSIILF	GCCACGATTTGAGGGACGAATC	55	WT: 1476 bp	This study
NSIIRR	ATCAGGATTAATGAAACGGACGC		NSII-CotA: 4400 bp	
NSILF	CGGAGCGCTGCTTTCTTGG	55	WT: 1665 bp	This study
NSIRR	CGGAGCGCTGCTTTCTTGG		NSI-CotA: 4679 bp	
PhycoBF	ATGCTAGATGCATTYRCCAARGTT	55	594 bp	This study
PhycoBR	TTAGGCWACNGCRGCAGCGGC	55	594 bp	This study

**Figure 1:**
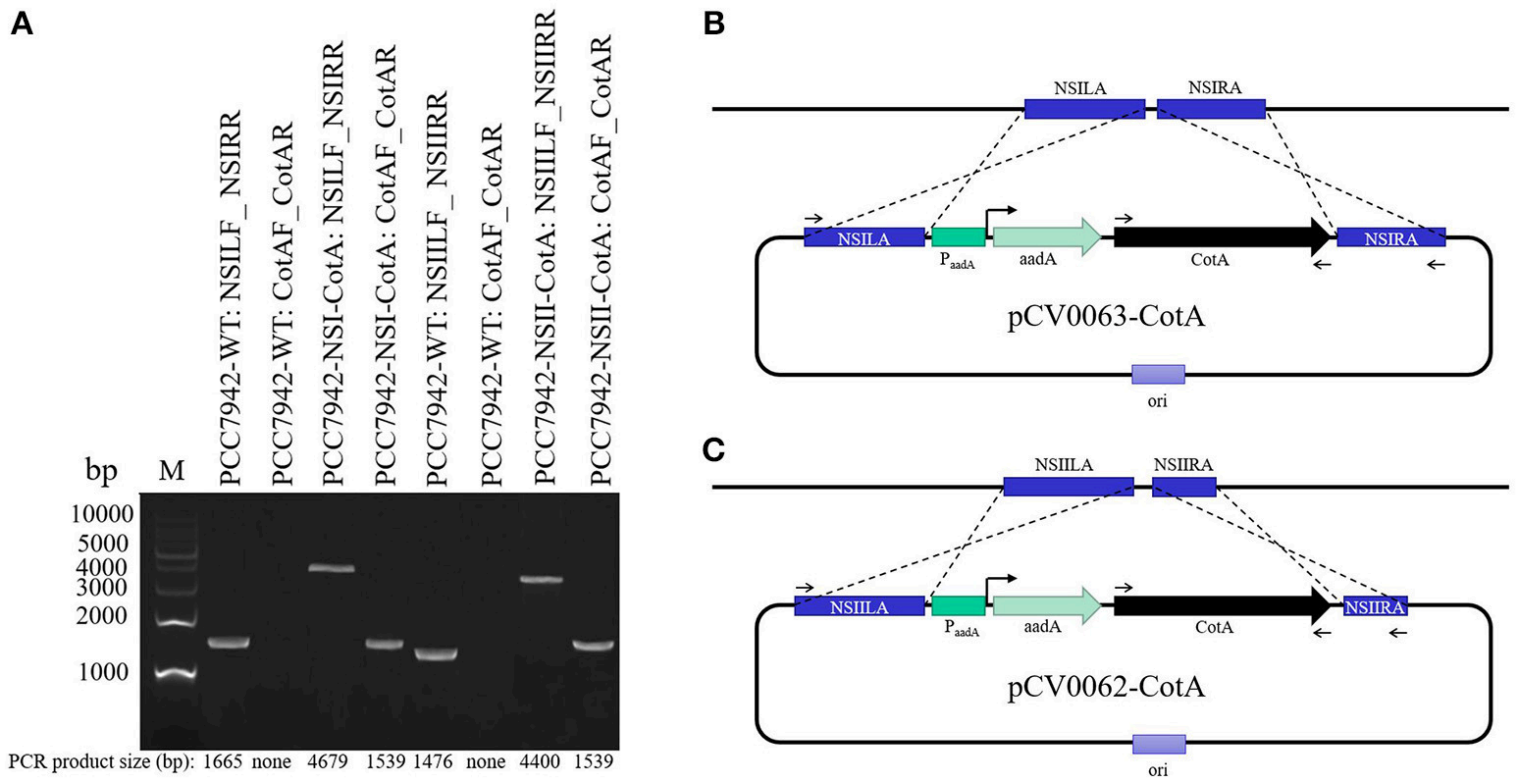
Analysis of successful transformants of PCC7942-NSI-CotA and PCC7942-NSII-CotA strains and the wild type PCC7942-WT strain. **(A)** PCR products confirming genomic arrangement of the transgenic strains and controls labeled in convention Strain ID: Forward primer ID_Reverse primer ID. Strain IDs and primer IDs can be found in Table 1, M, molecular weight marker. Actual PCR product sizes and sizes of markers are shown on the figure at the bottom and on the left, respectively. **(B)** Design features of the plasmid pCV0063 and the corresponding strain PCC7942-NSI-CotA. **(C)** design features of the plasmid pCV0062 and the corresponding strain PCC7942-NSI-CotA. Binding sites for primers from Table 1 are shown sections **(B,C)** of the figure.

The destination vectors pCV0063 and pCV0062 (Figure 1, Table 2) containing expression cassette for cytoplasmic expression under the control of constitutive promoter and targeting sequences integration toward NSI (genomic locus tag: SYNPCC7942_RS12675) and NSII (genomic locus tag: SYNPCC7942_RS00415) of *S. elongatus* PCC7942 genome (Figure 1A, Table 2) were assembled from individual parts with Cyanovector system (http://golden.ucsd.edu/CyanoVECTOR/). Parts deposited in NCBI with accession numbers KM017870, KM017923, KM017937 were used for construction of pCV0062 plasmid, whilst parts KM017870, KM017920, KM017937 were used for construction of pCV0063. Assembly was carried out using combination of blunt restriction enzymes ZraI and EcoRV (NEB, USA) and GeneArt® Seamless PLUS Cloning and Assembly Kit (Life Technologies, USA) essentially as described by Taton et al. (2014). Both destination vectors were confirmed by sequencing, digested with SwaI, and used for making expression vectors pCV0062-CotA and pCV0063-CotA. In brief, codon-optimized CotA gene amplified with Phusion *Pfu* DNA polymerase (NEB, USA) using primers CotA-veF, CotA-veR (Table 1) according to manufacturer's instructions. PCR products were purified with DNA Clean & Concentrator®-5 (Zymo Research, USA) and assembled with SwaI linearized vectors pCV0062 and pCV0063 using GeneArt® Seamless PLUS Cloning and Assembly Kit according to manufacturer's instructions.

**Table 2:** Plasmids and strains used in this study.

**Plasmid ID**	**Relevant characteristics**	**Reference**
pCV0062	Plasmid contains comprises: the chromosome integration site NSII for *Synechococcus elongatus* PCC7942; origin of replication for *E. coli* derived from pBR322 and an oriT site for conjugal transfer; aadA promoter and gene that confers spectinomycin and streptomycin resistance; the ccdB gene (toxic for commonly used *E. coli* cell lines), which can be taken out with the SwaI nuclease.	Taton et al., 2014
pCV0063	Plasmid contains comprises: the chromosome integration site NSI for *Synechococcus elongatus* PCC7942; origin of replication for *E. coli* derived from pBR322 and an oriT site for conjugal transfer; aadA promoter and gene that confers spectinomycin and streptomycin resistance; the ccdB gene (toxic for commonly used *E. coli* cell lines), which can be taken out with the SwaI nuclease.	Taton et al., 2014
pCV0062-CotA	pCV0062 plasmid digested with SwaI nuclease, with codon-optimized CotA laccase inserted in SwaI digested site	This study
pCV0063-CotA	pCV0062 plasmid digested with SwaI nuclease, with codon-optimized CotA laccase inserted in SwaI digested site	
Strain ID		
PCC7942-WT	*S. elongatus* PCC 7942 wild type	Collection Pasteur
PCC7942-NSI-CotA	*S. elongatus* PCC 7942 NSI: PaadA-aadA-PconII-CotA	This study
PCC7942-NSII-CotA	*S. elongatus* PCC 7942 NSII: PaadA-aadA-PconII-CotA	This study

Assembled plasmids pCV0062-CotA and pCV0063-CotA were transformed into *E. coli* DH5α (Tiangen, China), confirmed by sequencing, and cultures harboring these plasmids were grown in 150 ml LB medium supplemented with streptomycin (50 μg ml^−1^) overnight for large scale plasmid preparation. Large scale plasmid preparations were made with Plasmid Maxi Preparation Kit (GenStar, China).

### Natural transformation of *S. elongatus* PCC7942 and selection of laccase-harboring transformants

*S. elongatus* PCC7942 grown at logarithmic phase (OD_730_ 0.4–0.5) was harvested by centrifugation and washed twice with fresh BG11 medium. Optical density of cell suspension was adjusted to OD_730_ = 2.5 by resuspending the cell pellet with fresh BG11 medium. Then, at least 10 μg of plasmid DNA (pCV0062-CotA and pCV0063-CotA for each construct respectively) and 200 μl *S. elongatus* PCC7942 cell suspensions were mixed in 1.5 ml transparent microcentrifuge tubes. The tubes were cultivated without selective pressure for 24 hours. Tubes were occasionally shaken manually to prevent cells from settling. After 24 h of incubation cells were spread on a selective BG-11 medium supplemented with streptomycin (2 μg ml^−1^). One week later, streptomycin-resistant colonies that appeared on the plate were picked and transferred sequentially on BG11 plates supplemented with streptomycin (10 μg ml^−1^). Successful transformants have been repeatedly re-streaked on BG-11 containing increasing concentration of streptomycin. To achieve homogenous lines and integration of inserts to all copies of *S. elongatus* PCC7942 chromosome, cells were repeatedly re-streaked to fresh BG-11 medium containing increasing concentration of streptomycin (up to 50 μg ml^−1^) until PCR test with NSILF and NSIRR primers showed no secondary products of ~1.6 kb for Neutral site I construct (PCC7942-NSI-CotA strain), and NSIILF and NSIIRR primers showed no secondary products of ~1.5 kb for Neutral site II construct (PCC7942-NSII-CotA strain). Constructs were tested for residual vector backbone with vector specific primers and proved negative.

### Laccase activity assays

Laccase activity was determined in the cell-free culture medium, using typical substrates for determination of laccase activity: 2,2-azino-bis (3-ethylbenzothiazoline-6-sulfonate) (ABTS) or Syringaldazine (SYR) (Harkin and Obst, 1973; Childs and Bardsley, 1975). The oxidation of the substrate was detected by measuring the absorbance at 420 nm for ABTS (= 36,000 M^−1^ cm^−1^; Childs and Bardsley, 1975), and 530 nm for SYR (= 65,000 M^−1^ cm^−1^; Daroch et al., 2014). The typical reaction mixture (200 μl) contained 150 μl cell-free culture medium of either *S. elongatus* PCC7942 positive transformant or wild type, (grown under conditions described in culture conditions section Strains and Culture Conditions above) and 50 μl of 10 mM substrate. Reaction was incubated for 1 h at 30°C. Laccase activity was expressed in international units i.e., amount of laccase that oxidizes 1 μmol of substrate per minute. The absorbance at respective wavelengths was measured with a Multiskan Ascent spectrophotometer (Agilent, USA). All the assays were performed in triplicate. ABTS and SYR were purchased from Aladdin (China).

### Dye decolorization

Seven dyes were selected for decolorization using recombinant CotA-laccase produced with the PCC7942-NSII-CotA strain. Three classes of dyes were used: anthraquinonic- [Reactive Blue 19 (RB19) and Reactive Blue 4 (RB4)]; azo- [Reactive Black 5 (RB5), Acid Red 18 (AR18), Acid Red 27 (AR27), Acid Yellow 18 (AY18), Reactive Red 11 (RR11), and Reactive Orange 5 (RO5)], and an indigoid dye [Indigo carmine (IC)]. The reaction mixture (300 μl) contained 50-mM phosphate-citric acid (either pH 5.5 or 7.5), dye (final concentration 0.1 g/l), cell-free culture medium (100 μl, obtained after 10 days of cultivation in conditions described in section Strains and Culture Conditions) and ABTS mediator solution (concentration 0 or 10 mM). Reactions were incubated at 30°C for 7 days. Two types of controls were run in parallel at identical conditions. First control used wild-type *S. elongatus* PCC7942 cell-free culture medium grown under identical conditions, second control used sterile BG-11 medium. Degree of dye decolorization was determined by measuring residual absorbance at maximum absorption wavelength of the dye and expressed as percentage of decolorization. Decolorization assays were performed in triplicate. All dyes were purchased from Aladdin (China).

### RNA isolation and RT-PCR

Total RNA was extracted from 50 ml *S. elongatus* PCC7942 cultures at an OD_730_ of 0.3~ 0.5 using a Trizol (Sigma Aldrich) reagent. RNAse-free DNAse was used to remove genomic DNA. The reverse transcription reaction was carried out using SMARTScribe™ Reverse Transcriptase (Clonetech) and random primers. The resultant cDNA molecules were amplified by PCR using the following gene-specific primers: CotAF and CotAR (Table 1) for amplification of CotA and control primers PhycoBF and PhycoBR (Table 1) for amplification of the highly constitutively expressed C-phycocyanin beta-chain gene, which was used as a positive control. PCR products were analyzed by 0.8% agarose gel electrophoresis.

### SDS-PAGE and CotA laccase identification

Protein extraction procedure was followed by Xue et al. (2014). Sodium dodecyl sulfate-polyacrylamide gel electrophoresis (stacking gel 5%; resolving gel 12%) was performed according to the method of Laemmli (1970) using a Mini-Protean Tetra vertical electrophoresis system (Bio-Rad, USA). Gels were stained with Coomassie Brilliant Blue. Protein band corresponding CotA laccase was excised from the gel and sequenced at BGI (Shenzhen, China) with LC\MS\MS(Q-TOF).

## Results and discussion

### Construction of photosynthetic microbial cell factory for laccase production

In this work, we have successfully designed and constructed using synthetic biology approach recombinant strains of *S. elongatus* PCC7942 capable of expressing *B. subtilis* CotA laccase. Integrative vectors were designed and assembled from parts using Cyano-vector system developed by Taton et al. (2014). Recombinant strains were constructed using natural transformation of the strain with integrative plasmids targeting two neutral sites in cyanobacterial genome (NSI and NSII). Genetically fully segregated lines were obtained through increasing antibiotic selective pressure until all copies of genome contained desired inserts. To verify stable integration of codon-optimized CotA gene into neutral sites of the genome PCR tests were performed (Figure 1). The primers specific to both neutral sites of the genome were selected along (Table 1, Figure 1). Genomic DNA from the wild-type strain was used as a control. PCRs using genomic DNA (PCC7942-WT, PCC7942-NSI-CotA, PCC7942-NSII-CotA) with primer pairs flanking the neutral sites (Table 1) generated amplification products of desired sizes (consult the Figure 1 and Table 2 for details of actual sizes and primer positioning). That confirmed successful construction of both strains.

### Photosynthetic production of CotA laccase in *S. elongatus* PCC 7942

*S. elongatus* PCC7942 wild type and CotA-expressing strains (PCC7942-NSI-CotA and PCC7942-NSII-CotA) were grown in BG-11 medium containing streptomycin under constant illumination with fluorescent white light. Since BG-11 contains copper sulfate no additional copper supplementation to fulfill metal requirement of the enzyme was used. Simultaneously, indicator cultures of these strains in BG-11 medium containing 10 mM ABTS and SYR, were also established and monitored during 7 days of cultivation for colored product of ABTS and SYR oxidation, respectively. During 7 days of cultivation both transgenic strains oxidized both colorimetric substrates whilst wild type strain was unable to do so.

**Figure 2:**
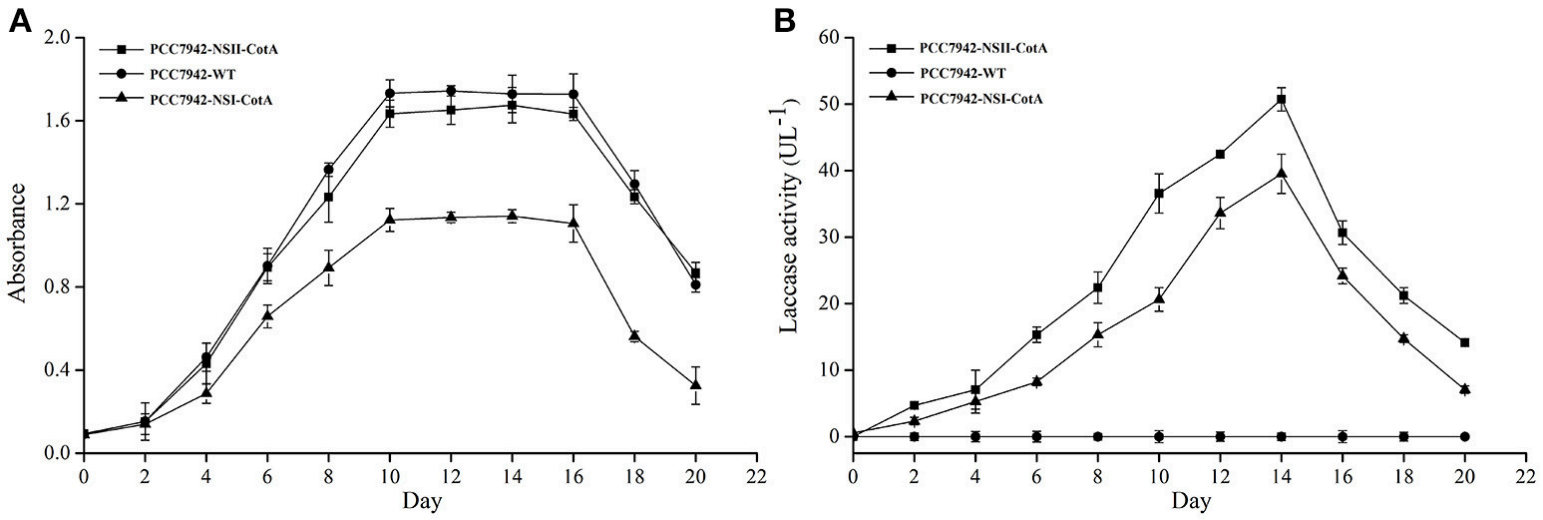
Cultivation of transgenic strains PCC7942-NSI-CotA and PCC7942-NSII-CotA in BG 11 growth medium: **(A)** growth curve of PCC7942-NSII-CotA (squares ■), PCC7942-NSI-CotA (triangles ▴) and PCC7942-WT (circles •); **(B)** laccase activity curve in cell-free culture medium of PCC7942-NSII-CotA (squares ■), PCC7942-NSI-CotA strain (triangles ▴), PCC7942-WT (circles •).

**Figure 3:**
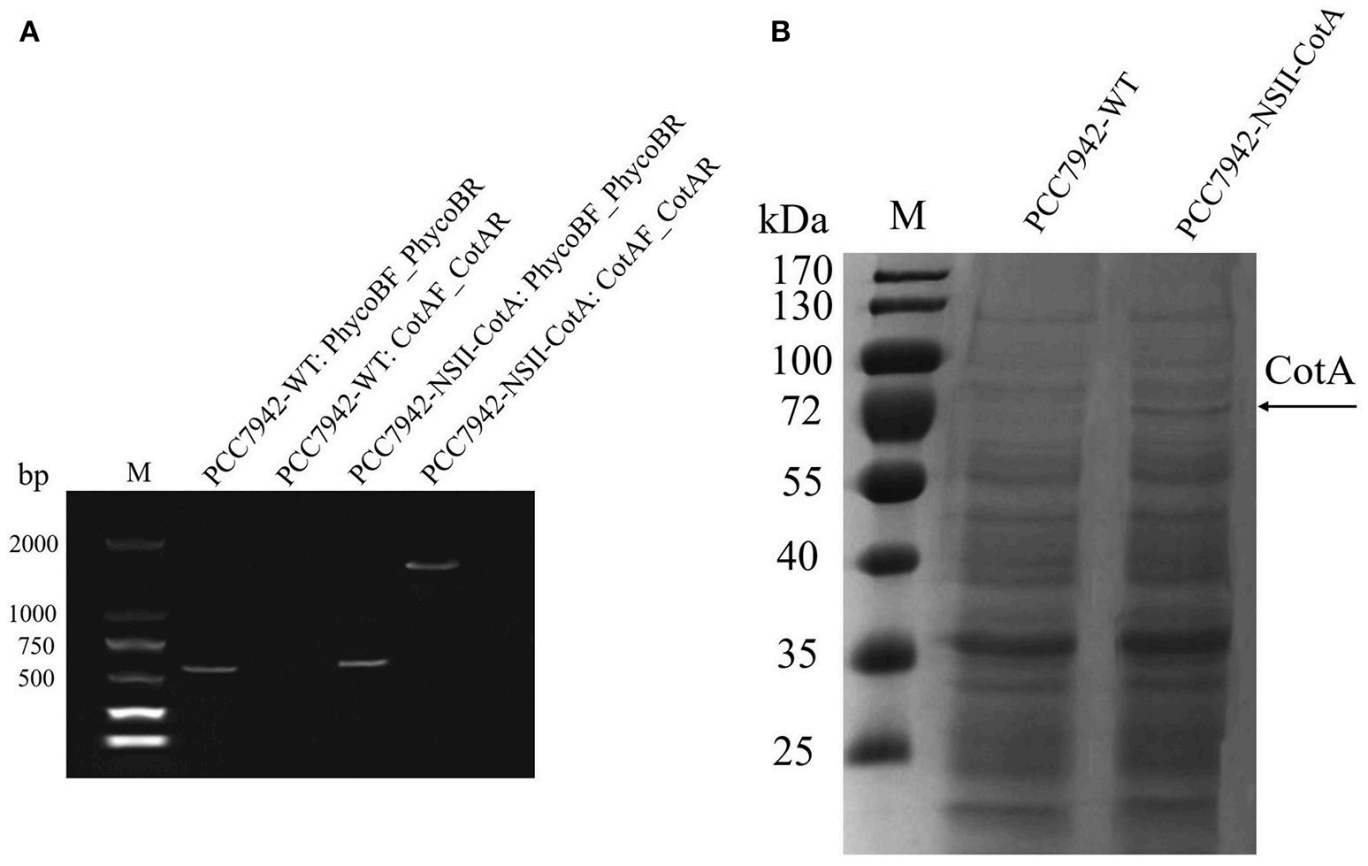
Expression analysis of the PCC7942-NSII-CotA and wild type PCC7942-WT: **(A)** RT-PCR products confirming expression of the laccase in strain PCC7942-NSII-CotA. Strains and controls labeled in convention Strain ID: Forward primer ID_Reverse primer ID. Strain IDs and primer IDs can be found in Table 1. Size of the CotA laccase gene is 1539 bp, and C-phycocyanin beta subunit gene, that was used as positive control, 519 bp. M, molecular weight marker. **(B)** SDS-PAGE of the CotA expressing *S. elongatus* PCC7942-NSII-CotA. Total cell extract of PCC7942-NSII-CotA and PCC7942-WT were fractionated by SDS/PAGE and stained with EZBlue™ Gel Staining Reagent. Lanes: PCC7942-WT: wild-type *S. elongatus* PCC7942; PCC7942-NSII-CotA: NSII-targeted, CotA-expressing transformant of *S. elongatus* PCC7942, M, molecular weight marker, Arrow indicates CotA band confirmed by peptide sequencing.

The recombinant CotA was constitutively expressed during cultivation of both *S. elongatus* PCC7942 as evidenced by similar trend of laccase activity and biomass production (Figures 2A,B). The growth rate of PCC7942-NSI-CotA was lower than the wild type *S. elongatus* PCC 7942 whilst PCC7942-NSII-CotA had similar growth rate (Figure 2A). Interestingly, laccase activity of recombinant cyanobacteria could be detected after 2 days of cultivation in the growth medium and increased along with cyanobacteria growth until day fourteenth. This was despite the enzyme not being targeted for extracellular export with known cyanobacterial signal peptide. That may suggest that CotA laccase may undergo non-classical secretion or other signal peptide independent secretion (Götz et al., 2015), like many other *B. subtilis* proteins (Bendtsen et al., 2005), or that it accumulates in culture medium for prolonged period from leaky or lysed cells showing good stability characteristic in the absence of buffering. Laccase activity was not detected in wild type *S. elongatus* PCC7942 cell-free culture medium. When cyanobacterial growth reached stationary phase, the PCC7942-NSII-CotA laccase activity rose gradually until reached 50.73 U l^−1^. The laccase activity of PCC7942-NSI-CotA was relatively lower than that of the other strain (Figure 2B). These results indicate that genomic integration at neutral site II is more suitable for expression of laccase enzyme than its insertion into neutral site I. This is interesting since it is generally assumed that both of so-called neutral sites used for gene “knock ins” in *S. elongatus* PCC7942 are equally suitable for transgene insertion. Our results indicate that it does not have to be the case with all genetic engineering efforts, and it may be worthwhile to test multiple integration sites for each of the construct to test its effects for the host strain. As a result, construct targeting neutral site II PCC7942-NSII-CotA was selected for subsequent experiments. To determine whether the CotA transformant can express the integrated CotA laccase gene, we analyzed the transcription of CotA by reverse transcriptase (RT)-PCR (Figure 3A). DNA-free total RNA was extracted from the PCC7942-NSII-CotA culture, and first-strand cDNA was synthesized using random primers. The same procedure was used to obtain the wild type *S. elongatus* PCC7942 cDNA, which served as a control. The CotA -specific primers (Table 1) amplified a fragment of the expected size from cDNA isolated from the pCV0062-CotA-PCC7942 transformant, no product was obtained from the wild-type cDNA. Constitutively expressed under phototrophic growth C-phycocyanin beta chain gene (*cpcB*) product (519 bp) (Sawaki et al., 1998) served as positive control in both experiments and was successfully amplified from both CotA-expressing constructs and the wild type cDNA. Total cell extract protein from the PCC7942-NSII-CotA strain and wild-type *S. elongatus* PCC7942 was analyzed for CotA-laccase expression using SDS-PAGE. A 65 kDa band was present in total cell extract protein from the CotA transformant strain but not in that from the wild type (Figure 3B). The band was gel excised, peptide sequenced by LC\MS\MS(Q-TOF), and the sequence was compared with CotA laccase protein sequence deposited in database with accession number BAA22774.1. Peptide sequencing result revealed 100% sequence match and sequence coverage of 85%. Closer look at the ratio of intra- and extracellular fractions reveals that the majority of laccase was intracellular (specific activity 229 U_ABTS_ ${\text{mg}}_{\text{prot}}^{-1}$) whilst the specific activity of extracellular fraction was about 50% smaller (specific activity 128 U_ABTS_ ${\text{mg}}_{\text{prot}}^{-1}$) than that of intracellular one. This suggests that whichever of the two potential mechanisms i.e. non-classical secretion or cell leakiness resulted in the laccase transfer to the culture medium there is significant room for improvement of the protein production yields in the future.

### Dye decolorization by *Synechococcus*-expressed laccase

**Figure 4:**
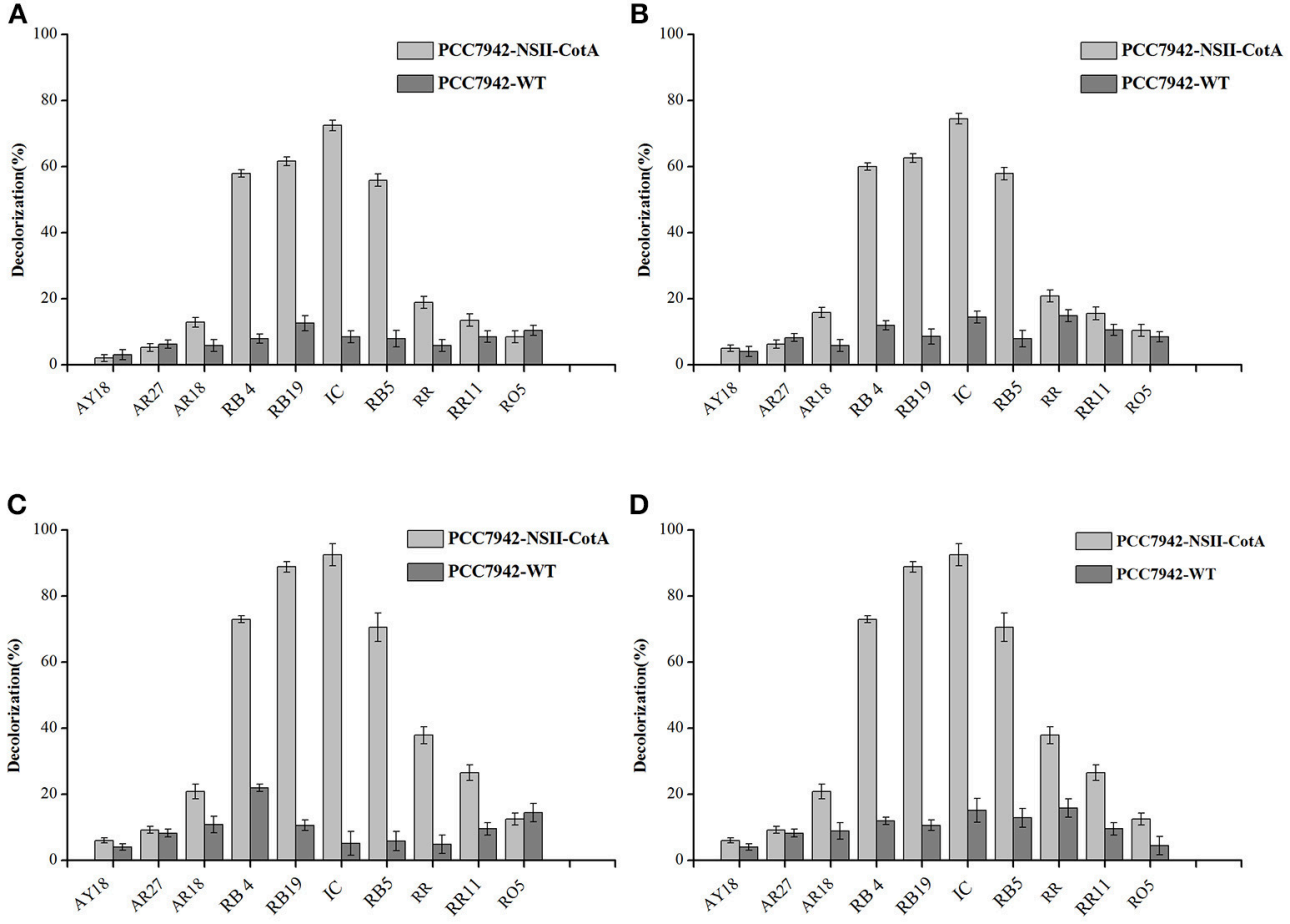
Decolorization of tested dyes (0.1 g/l) by cell-free culture medium containing CotA laccase produced by the recombinant cyanobacterium PCC7942-NSII-CotA (light gray bars) and by the cell-free culture medium of the control strain PCC7942-WT (dark gray bars) during 7 days of incubation. Following decolorization conditions were used: **(A)** No ABTS mediator in 0.05M phosphate-citric acid buffer (pH 5.5), **(B)** 10 mM (final) ABTS mediator in 0.05M phosphate-citric acid buffer (pH 5.5); **(C)** No ABTS mediator in 0.05M phosphate-citric acid buffer (pH 7.5); **(D)** 10 mM ABTS mediator in in 0.05M phosphate-citric acid buffer (pH 7.5). Abbreviations: Reactive Blue 19 (RB19); Reactive Blue 4 (RB4); Reactive Black 5 (RB5), Acid Red 18 (AR18), Acid Red 27 (AR27), Acid Yellow 18 (AY18), Reactive Red 11 (RR11) Reactive Orange 5 (RO5), and Indigo carmine (IC).

We have evaluated the ability of transgenic cyanobacteria-produced laccase for decolorization of several important industrial dyes belonging to: anthraquinonic- azo- and indigoid groups by transgenic cyanobacterial cultures over 7 days. Two variants of experiment were performed: effect of pH and use of redox mediator (ABTS). Four experimental conditions were tested: pH 5.5 with ABTS, pH 5.5 without ABTS, pH 7.5 with ABTS and pH 7.5 without ABTS. Results of decolorization experiment carried out during 7 days of incubation are summarized in Figure 4, results of control experiments, that utilized PCC7942-WT are presented on the same figure. Although the control strains show some degree of decolorization it is evident that the culture liquid from CotA-expressing strain yields significantly higher degree of decolorization in majority of dyes tested.

At more acidic conditions, all dyes exhibited degrees of decolorization lower than 80%. The dyes with highest degrees of decolorization without use of ABTS were: Indigo carmine (72.52%), Reactive Blue 19 (61.63%), Reactive Black 5 (55.89%), and Reactive Blue 4 (58.01%). Similar results were observed during decolorization of these dyes with the use of ABTS as a mediator (Figures 4A,B) indicating no effect of the mediator for efficiency of dye decolorization. At more alkaline pH 7.5 the degrees of decolorization were higher (Figures 4C,D). The highest degree of decolorization (92.52%) under pH 7.5 without ABTS was achieved for Indigo carmine followed by Reactive Blue 19 (88.89%), Reactive Black 5 (71.64%), and Reactive Blue 4 (73.01%). Similarly, addition of ABTS did not seem to have any effect on the degrees of decolorization at higher pH either. This is an important development since mediators are generally expensive and especially non-natural mediators like ABTS are cost-prohibitive in any bioremediation reactions like dye decolorization. Our results show that optimal pH for CotA-laccase decolorization using our strain was pH 7.5 rather than pH 5.5, typical for other laccases used such applications. For example many fungal laccases exhibit optimal performance for dye decolorization in the acidic range of pH (Abadulla et al., 2000; Baldrian, 2004; Camarero et al., 2005; Rodríguez Couto et al., 2005; Zille et al., 2005; Pogni et al., 2007). Such pH is usually outside the range of protein stability (Baldrian, 2004) and results in quick inactivation of biocatalyst. Moreover, a removal study like this one would require both host cell and protein to be stable at low pH whist maintaining active growth this is unlikely using most cyanobacteria that prefer more alkaline growth conditions. A combination of protein and expression host with higher pH of stability, activity and growth is better suited for this application. Previous studies of CotA laccase have shown that dye decolorization can be carried out in higher pH than those preferred by fungal laccases. Wang et al. carried out decolorization of indigo carmine at pH 10.0 whilst, pH 8–9 was used for successful decolorization of azo dye Sudan Orange G (Pereira et al., 2009; Wang et al., 2015). In our study CotA-transformed *Synechococcus* strain was able to decolourise three types of dyes (anthraquinonic-, azo-, and indigoid) to a high extent (above 80%) making it a potentially useful broad-spectrum biodegradation agent.

**Figure 5:**
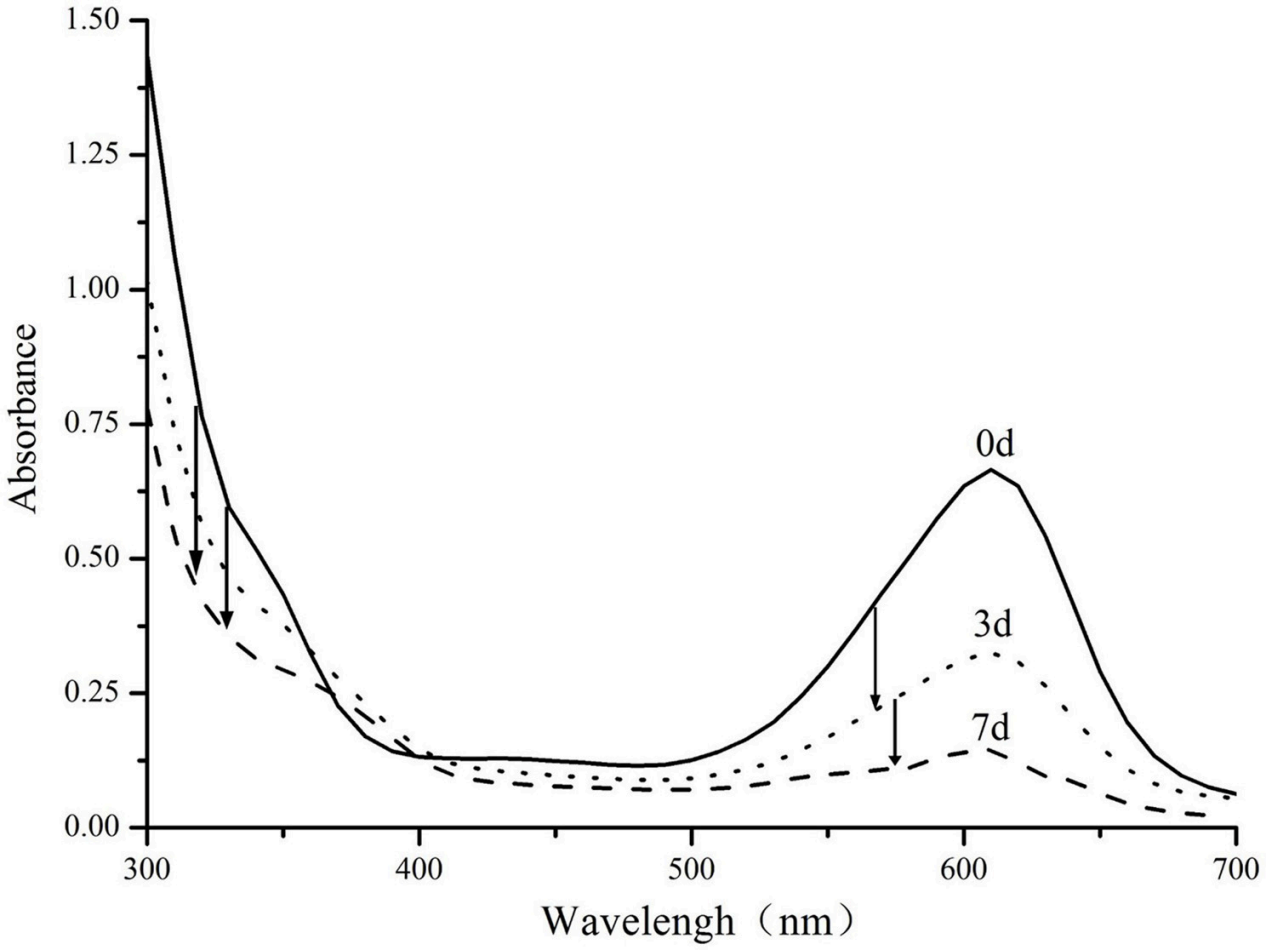
UV–Vis spectra of Indigo carmine (0.1 g/l) biodegraded by PCC7942-NSII-CotA produced recombinant laccase at times: 0, 3, 7 days. T = 30°C, pH = 7.5 Arrows indicate characteristic decreases in absorbance throughout the decolorization experiment.

Enzymatic biodegradation of Indigo carmine has been studied in more detail (Figure 5). Analysis of the absorption spectrum reveals that absorption peaks decrease proportionally throughout the degradation process and no major colored product is generated in the process. There is a however a minor peak between 350 and 380 nm which is formed as a result of decomposition. The exact mechanism of the degradation is likely to follow previously reported decolorization of indigo carmine with other laccases, including those of genus *Bacillus* (Wang et al., 2017). Previous reports suggest that the dye is likely to be degraded through formation of isatin which is further decomposed into anthalinic acid (Campos et al., 2001; Li et al., 2015; Wang et al., 2017). This route is also supported by the small absorbance band in near UV region that was previously observed in both anthranilic acid (Melad and Esleem, 2015) and our decomposition products.

## Conclusions

In the present work, we have constructed two vectors, each targeting different neutral site of the genome, for the expression of industrially relevant CotA-laccase using the modular vector system. The two strains did not exhibit similar growth fitness, which may suggest that neutral site II of the *S. elongatus* PCC 7942 is better suited for the expression of the transgene than neutral site I is. Interestingly, CotA laccase accumulated in the culture medium of the two transgenic strains despite the lack of clear signal peptide in laccase sequence. The exact reasons of this accumulation will be elucidated in the future. Culture medium-accumulated laccase was used for the degradation of an array of textile dyes of different chemical structures without the need for a mediator at pH 7.5 which is suitable for cultivation of this cyanobacterium. Laccase was the most effective in the degradation of an important dye indigo carmine, most likely to anthranilic acid analogously to other laccases. This process can be a promising alternative to conventional treatments such as adsorption and chemical oxidation due to its eco-friendliness and potentially low costs.

## Author contributions

YML and MD designed the study. YML, JH, and YFL prepared materials and accomplished experiments. YML, YL, and JT analyzed data and discussed results. YML wrote the manuscript. JC and MD offered revisions and approved the final manuscript.

### Conflict of interest statement

The authors declare that the research was conducted in the absence of any commercial or financial relationships that could be construed as a potential conflict of interest.
